# The effect of body-conforming passive wearable device with knee flexion taping on dynamic knee stability

**DOI:** 10.1017/wtc.2025.10022

**Published:** 2025-08-26

**Authors:** Sung-Jin Park, Seongok Chae, Hyung-Soon Park

**Affiliations:** Department of Mechanical Engineering, Korea Advanced Institute of Science and Technology, Daejeon, Republic of Korea

**Keywords:** passive wearable device, knee flexion taping, injury prevention, dynamic stability, biomechanics

## Abstract

Passive wearable devices are widely used for fitness and have also become fashionable. There is increasing interest in adding functionality, such as knee stability, to these compact devices, which are more convenient for daily wear than separate devices like braces or exoskeletons. This study designed and assessed flexion taping passive wearable devices (FTPW). The design emphasized providing adequate flexion moment capacity and controlling varus/valgus movement to prevent knee injuries. In this research, 20 healthy women performed single leg drop (SLD) and step-up (SU) tests with and without muscle fatigue. Knee joint angle, muscle activation, metabolic cost, and blood flow were measured across FTPW, passive wearable devices without flexion taping (PW), and control shorts (Ctrl). In the SLD test after muscle fatigue, FTPW produced a significantly larger knee flexion angle during landing. In the SU test, before and after fatigue, knee varus angle was notably higher with FTPW. Additionally, FTPW showed reduced knee flexor fatigue, indicated by smaller median frequency shifts, and improved blood flow compared to PW. No significant differences in respiratory exchange ratio were detected among the three conditions. Overall, FTPW demonstrated strong potential to enhance knee kinematics, muscle activation, and blood flow, pointing to benefits for both performance improvement and injury prevention. By delivering focused support in a compact format, FTPW may serve as an innovative passive wearable solution that supports daily movement, comfort, and daily activities. This emphasizes the device’s promise as an alternative to bulkier knee aids, merging style and functionality effectively.

## 1. Introduction

The knee joint is the most commonly injured body part when the foot contacts the ground (Ford et al., 2003). Especially in the case of women, injuries appear repeatedly and chronically for the following reasons: persistent and abnormal forward movement of the tibia in relation to the femur (Kernozek et al., 2005; Arden and Nevitt, 2006), lower value of the hamstring/quadriceps strength ratio (Holcomb et al., 2007), and large knee valgus angle (Myer et al., 2006).

In particular, previous studies reported that overly small knee flexion and excessively large knee valgus angle may be leading causes of knee injuries (Chappell et al., 2002; Kernozek et al., 2005). Additionally, imbalanced muscle reaction forces, such as excessively high quadriceps muscle force and low hamstring muscle contraction force, can contribute to knee injuries during landing. Of particular concern is the significant gender disparity in noncontact knee injury rates (McLean et al., 2005). For instance, in sports such as basketball, soccer, team handball, and daily activity, women are reported to experience knee injuries two to seven times more frequently than men. The sports medicine community has been actively trying to change this trend, with new injury prevention strategies being actively and extensively studied (Alentorn-Geli et al., 2009).

To address these challenges, various wearable devices, such as knee braces, have been applied to increase knee stability and to prevent knee injuries (Yang and Lim, 2014). For example, in an epidemiological study of American football players, wearing a knee brace reduced the injury rate by ~2.7 times (Rishiraj et al., 2009). It was reported that knee braces were effective in keeping the knee from excessive flexion or extension during exercise and decreasing the load applied to the knee by adjusting hamstring muscles and gastrocnemius muscle activity involved in knee flexion (Wang et al., 2023). Furthermore, using the knee valgus brace increases the knee’s mediolateral stability and reduces the risk factors (Ramsey et al., 2007). However, it has been noted that individuals without knee injuries found knee braces inconvenient to carry or wear, often expressing discomfort due to tightly strapped bands.

Another approach to prevent injury is to use clinical kinesio tape made of adhesive flexible materials that do not hinder movements in daily activities and can be applied directly to the skin. However, its practical limitations, such as high cost, potential for skin irritation, and the necessity of professional application, restrict its widespread usability, particularly for nonprofessional users in daily settings. Despite these benefits, taping is not easily applied by individuals in daily life due to the high price, potential skin troubles, and the need for professional expertise for accurate application.

On the other hand, interest in passive wearable devices has been increasing recently, particularly due to their potential to integrate advanced functionalities, such as elastic taping, which aligns with the goals of enhancing both performance and injury prevention in daily activities. The functional effects that occur after wearing passive wearable devices are broadly divided into biomechanical and physiological aspects. The biomechanical effects of passive wearable devices are reduction of muscle tremors and repetitive jumping power enhancement (Kramer et al., 2018). The physiological effect helps a quick recovery after exercise by increasing blood flow and reducing fatigue through muscle oxygenation (Troynikov et al., 2010; Joyner and Casey, 2015; McNeil et al., 2015). Because of these effects, not only athletes but also recreational sports participants can wear passive wearable devices for improving performances.

In addition to this, there is an attempt to enhance the performance of passive wearable devices by adding elastic taping. While such clothing has the disadvantage of providing less assistance compared to assistance robots, it has the advantage of being lightweight and easy to wear. For instance, when wearing a passive wearable device with added taping to assist knee extension, the execution of sit-to-stand was performed more efficiently (Lee et al., 2020). Therefore, if the intended direction is achieved and a subtle yet continuous assisting force is applied in daily life, it is expected to have a positive effect on injury prevention in activities of daily living, similar to performance enhancement.

The injury prevention effects of wearing different types of clothing are evaluated using the single leg drop (SLD) test and the step-up (SU) test. The SLD test involves movements that expose individuals to injury risks in sports activities and can be used to assess knee joint stability. Particularly during landing, significant differences in the forces acting on the anterior cruciate ligament (ACL) are observed, depending on the knee flexion angle (Laughlin et al., 2011). The SU test, utilized in rehabilitation programs (Paz et al., 2016), measures the range of motion in knee flexion. This study will examine the effect of assisting force-induced post-fatigue on knee joint movement via the SU test.

This study introduces a novel passive wearable device design, the flexion taping passive wearable devices (FTPW), which uniquely integrates elastic taping to provide dynamic support for the knee joint. By addressing limitations of traditional braces and kinesio tape, the FTPW offers a lightweight and variable solution to enhance knee stability, particularly under fatigue-induced conditions. The FTPW’s potential applications span athletic performance enhancement, injury prevention in high-risk sports, and rehabilitation for individuals with knee instability. To evaluate its efficacy, this study investigates the effects of fatigue on kinematics, muscle activation, blood flow, and respiratory exchange ratio (RER) using the SLD and SU tests, simulating real-world conditions faced by athletes and women. To verify the effectiveness of the proposed passive wearable device, we compared the effect of wearing FTPW devices with passive wearable devices without flexion taping (PW) and loose shorts without any compression (Ctrl).

## 2. Materials and methods

### 2.1. Design of passive wearable devices with/without knee flexion taping

**Figure 1.**
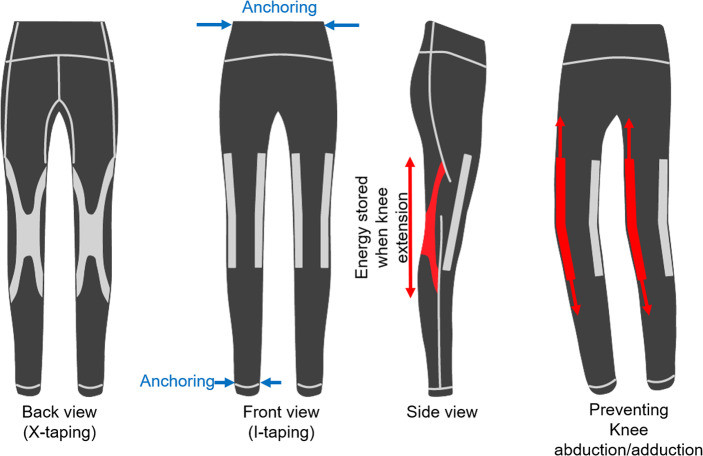
Design overview of FTPW. X-taping for knee flexion was applied to the back of the knee, and I-taping for preventing knee abduction/adduction was applied to the medial/lateral sides of the knee, respectively. To prevent slipping during motion, bands were used to anchor the garment to the ankle and waist.

The PW was created as a body-conforming device that provides uniform compression. Building on this foundation, the FTPW incorporates two taping layouts to deliver task-specific support (Figure 1). An X-pattern added across the back of the knee stores elastic energy during knee extension and assists flexion by passing over the hamstrings and gastrocnemius. An I-pattern is added along the medial and lateral collateral ligament regions to limit excessive knee abduction and adduction. Thus, the FTPW retains the compression of the PW while adding functional elastic taping for flexion assistance and frontal-plane stabilization.

X-taping (370 N/m) required elasticity to facilitate knee flexion, and for I-taping (500 N/m), higher stiffness than X-taping was set to prevent excessive knee abduction/adduction. Anchoring components were placed at the garment’s waist and ankle to avoid slipping of the edge, which decreases the amount of elastic force created by the taping. The stiffness of the taping was calculated as the ratio of the increase in length in response to applied weight. The pressure exerted by both the FTPW and PW on the pelvis and ankle was determined by comparing the wearer’s waist circumference to the device’s circumference before and after wearing, following a method described by Lee et al. (2020). The measured pressure was 3.5 kPa at the pelvis and 2.9 kPa at the ankle for both conditions. The total weight of the FTPW and PW was 150 g.

### 2.2. Experimental protocol

Twenty healthy women without a history of musculoskeletal diseases and cardiovascular diseases participated in the study (age: 38.6 ± 5.3 years, weight: 63.3 ± 9.3 kg, body mass index: 23.6 ± 3.6; mean ± standard deviation [SD]). Informed consent was obtained from all subjects using the form approved by the Institutional Review Board of Korea Advanced Institute of Science and Technology (KH2021–036). An experimental protocol was designed to evaluate the effects of FTPW on kinematics, muscle activation, and RER during a fatigued state.

All subjects were tested while wearing three different types of garments: FTPW, PW, and Ctrl. To prevent carryover effects, a randomized three-way crossover design was employed. To minimize the effects of fatigue and learning between experimental sessions, the test interval was set to 1 week.

**Figure 2.**
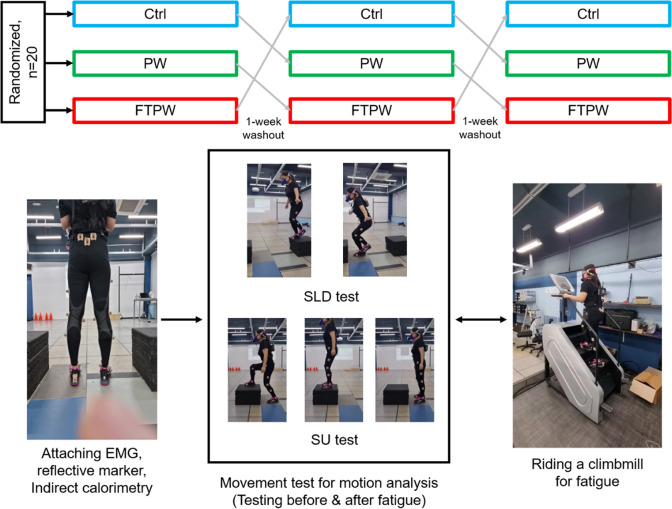
The experimental procedure in this study. All participants completed three trials, at a 1-week interval. SLD/SU tests were performed before and after fatigue riding the Climbmill.

Changes in kinematics due to fatigue were evaluated using the SLD and SU tests (Figure 2). Twenty-one reflective markers were placed on lower extremity landmarks based on a Plug-in Gait marker system (Kadaba et al., 1990). In the SLD test, subjects stood on their dominant leg on a 30 cm high box and were instructed to drop to a spot on the force plate (Advanced Mechanical Technology, Inc., MA, USA) 30 cm in front of the box without making any additional compensatory movements after landing. For the SU test, participants stepped up onto a 30 cm box with their dominant foot and then stepped down again with the same dominant foot after planting both feet at the top of the box. Both the SLD and SU tests were repeated three times before and after the fatigue protocol. To maintain the effect of fatigue, these tests were performed within 30 s of completing the fatigue protocol.

The fatigue protocol involved the use of a Climbmill (Shandong Minolta Fitness Equipment Co., Ltd, Ningjin Town, China), a gradual fatigue-inducing machine that targets the lower limbs. In this study, six electromyographic (EMG) sensors were attached to vastus medialis (VM), vastus lateralis (VL), semitendinosus (ST), biceps femoris (BF), medial gastrocnemius (MGT), and lateral gastrocnemius (LGT). We used an EMG sensor covered by cushions to reduce pressure increase. Subjects wore indirect calorimetry (Cosmed K5: Cosmed, Rome, Italy) to calculate the metabolic cost during the fatigue protocol. This machine had 30 cm high steps and operated at speeds ranging from 24 to 164 steps/min. Subjects climbed at an increased speed of 20 steps/min every 3 min after the exercise began. The fatigue protocol continued until the subject’s heart rate reached 80% of maximum heart rate, which is the criterion for fatigue in this experiment. The heart rate meter employed in this study was the Polar OH1 (Polar Electro Oy, Kempele, Finland). The maximum heart rate was calculated using the formula: HRmax = 220 − age, according to the preceding paper (Mj, 1957).

Before testing, subjects rested for a minimum of 15 min to allow their heart rates to stabilize. Once heart rates reached a stable state, blood flow velocity in the common carotid artery was measured for 10 s using an ultrasound blood flow meter. The blood flow velocity was measured using ultrasonography (SONON 300 L, Healcerion Co., Ltd, Seoul, Republic of Korea) at a sampling rate of 10 Hz. The measurement was performed three times to obtain an accurate reading.

### 2.3. Data analysis

For motion analysis, the VICON NEXUS system (Oxford Metric Ltd, Oxford, UK) consisting of 12 cameras runs at a sampling rate of 100 Hz. Following data collection from the SLD and SU tests, the knee joint angle, knee joint moment, knee joint force, and ground reaction force (GRF) data were processed using Visual 3D (C-motion Inc., Germantown, MD). The knee joint angle was calculated as the angle of the local coordinate system of each femur and tibia (Wu et al., 2002). To standardize the timing of each test, the data were normalized as percentages of the task period. During the SLD test, the starting point of the motion was set at 0% and the time point when the dominant foot touched the floor was set as 25%. The remaining time after landing was adjusted to match 100% or three times the length of the landing time. During the SU test, the point when the dominant foot began to move on the floor was set to 0% and the point when the opposite foot touched the floor was set to 100%. When normalized, the motion phases could be approximately divided into the dominant foot stepping onto the box (0–25%), the opposite foot stepping onto the box (26–50%), the dominant foot stepping down to the floor (51–75%), and the opposite foot stepping down to the floor (76–100%).

For EMG analysis, the Trigno Wireless EMG system (Delsys Inc., Boston, MA, USA) was employed at a sampling rate of 2,000 Hz. A 30-s window of muscle activity at both the start and end of the fatigue protocol was used when calculating the median frequency. A 50–500 Hz bandpass filter was applied during data processing. Data from the start and end of the fatigue protocol were used to divide the power spectral density into two regions containing equal amounts of power via fast Fourier transform (Hsu et al., 2020). This is the midpoint, a medium frequency used to evaluate the start and end of the fatigue protocol.

RER is calculated as the ratio of the produced CO_2_ volume to the consumed O_2_ volume. Average RER values during the final minute of the fatigue protocol were computed to discern differences among the various experimental conditions. Blood flow rate (BFR) was calculated by multiplying the cross-sectional area of the common carotid artery and the average velocity of the blood flow, as specified in a previous study (Schorer et al., 2017).

To assess participants’ subjective experiences while wearing the devices, a post-experiment subject assessment was conducted for the PW and FTPW conditions. The Ctrl condition was excluded from the survey because it provided neither compression nor structural support and served only as a baseline condition. The survey used a 5-point Likert scale to assess comfort, pressure sensation, and the general feeling of wearing the device, with a total of 14 inquiries (Chyung et al., 2017).

### 2.4. Statistical analysis

The sphericity of the data was assessed using Mauchly’s sphericity test. In cases where Mauchly’s sphericity test yielded a positive result, repeated-measures analysis of variance (ANOVA) with Tukey’s post-hoc analysis was employed to determine significant differences between experimental conditions. Differences in subjective assessments between the FTPW and PW conditions were examined with paired *t*-tests. Statistical analysis for median frequency difference, RER, BFR, peak vertical GRF (vGRF), and subject assessments between conditions was performed using SPSS Statistics 26 (IBM Corp., New York, USA). To determine the statistical significance of the knee joint angle, joint moment, and joint power at each time point during the SLD and SU tests, Statistical Parametric Mapping analysis was employed (Pataky et al., 2013).

## 3. Results

**Figure 3.**
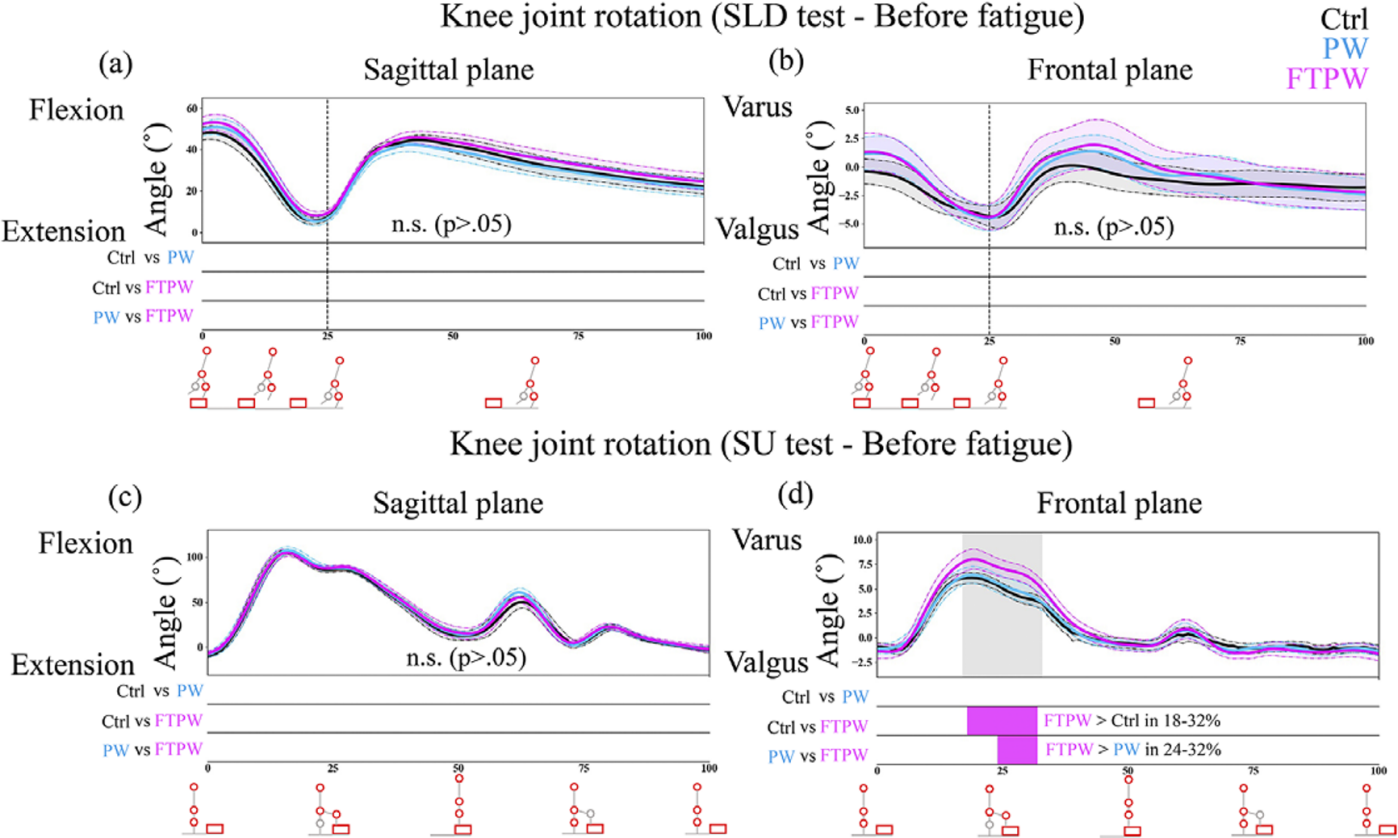
Knee joint rotation during the SLD/SU test (a, b/c, d) before the fatigue protocol. The black, blue, and magenta lines represent Ctrl, PW, and FTPW, respectively. Error bars mean standard error. The gray-shaded area corresponds to sections where the repeated-measures ANOVA results indicate a statistically significant difference (*p* < .05). Below each graph, bars compare group differences at each time point: Ctrl versus PW, Ctrl versus FTPW, and PW versus FTPW. The color of bars corresponds to the group with larger values.

**Figure 4.**
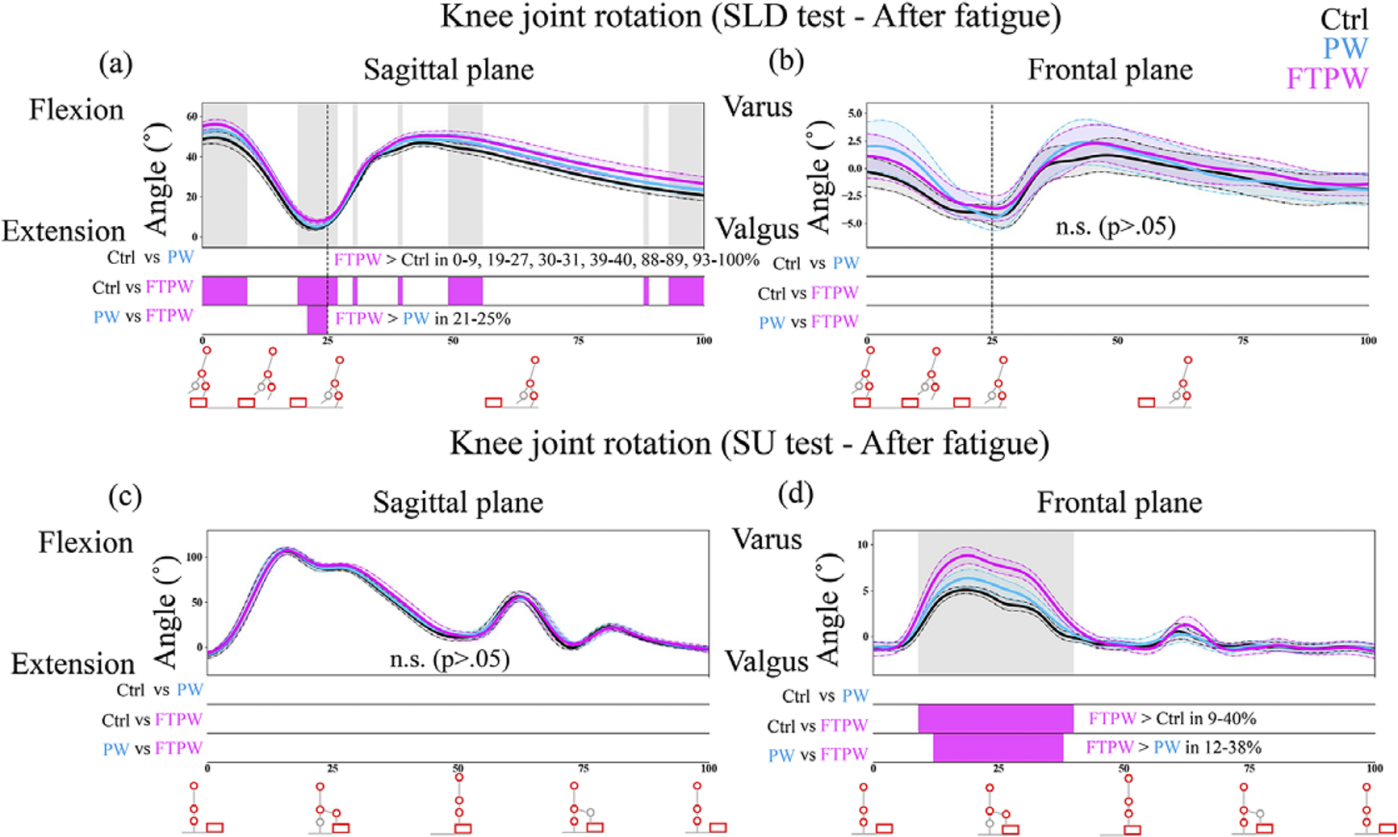
Knee joint rotation during the SLD/SU test (a, b/c, d) after the fatigue protocol. The black, blue, and magenta lines represent Ctrl, PW, and FTPW, respectively. Error bars mean standard error. The gray-shaded area corresponds to sections where the repeated-measures ANOVA results indicate a statistically significant difference (*p* < .05). Below each graph, bars compare group differences at each time point: Ctrl versus PW, Ctrl versus FTPW, and PW versus FTPW. The color of bars corresponds to the group with larger values.

Regarding kinematic analysis, before fatigue (Figure 3), there were no significant differences in sagittal/frontal plane kinematics during the SLD test (Figure 3b, 3c). However, during the SU test, knee varus was significantly greater in FTPW than PW and Ctrl when stepping up (Figure 3d). There were no significant differences in the sagittal plane kinematics (Figure 3c). In the kinematic analysis after fatigue (Figure 4), the knee flexion angle at landing was significantly greater in FTPW than PW and Ctrl during the SLD test (Figure 4a). Additionally, during the SU test, knee varus was significantly greater in FTPW than PW and Ctrl when stepping up (Figure 4d). No significant differences were found in frontal/sagittal plane kinematics during the SLD/SU test, respectively (Figure 4b, 4c).

In the kinetic analysis, knee joint flexion moment (Supplementary Figure S5A), superior force acting on the knee joint (Supplementary Figure S5E), and peak vGRF (Supplementary Figure S6) were all significantly lower in the FTPW than in Ctrl.

In the kinematic analysis, dividing the subjects into groups of 10 each based on the varus knee alignment of their dominant leg’s tibiofemoral angle (low varus knee; −9.83 ± 1.51°, high varus knee; −7.21 ± 1.29°; mean ± SD), both groups demonstrated congruence in the SLD/SU test (Supplementary Figures S1–S4).

**Figure 5.**
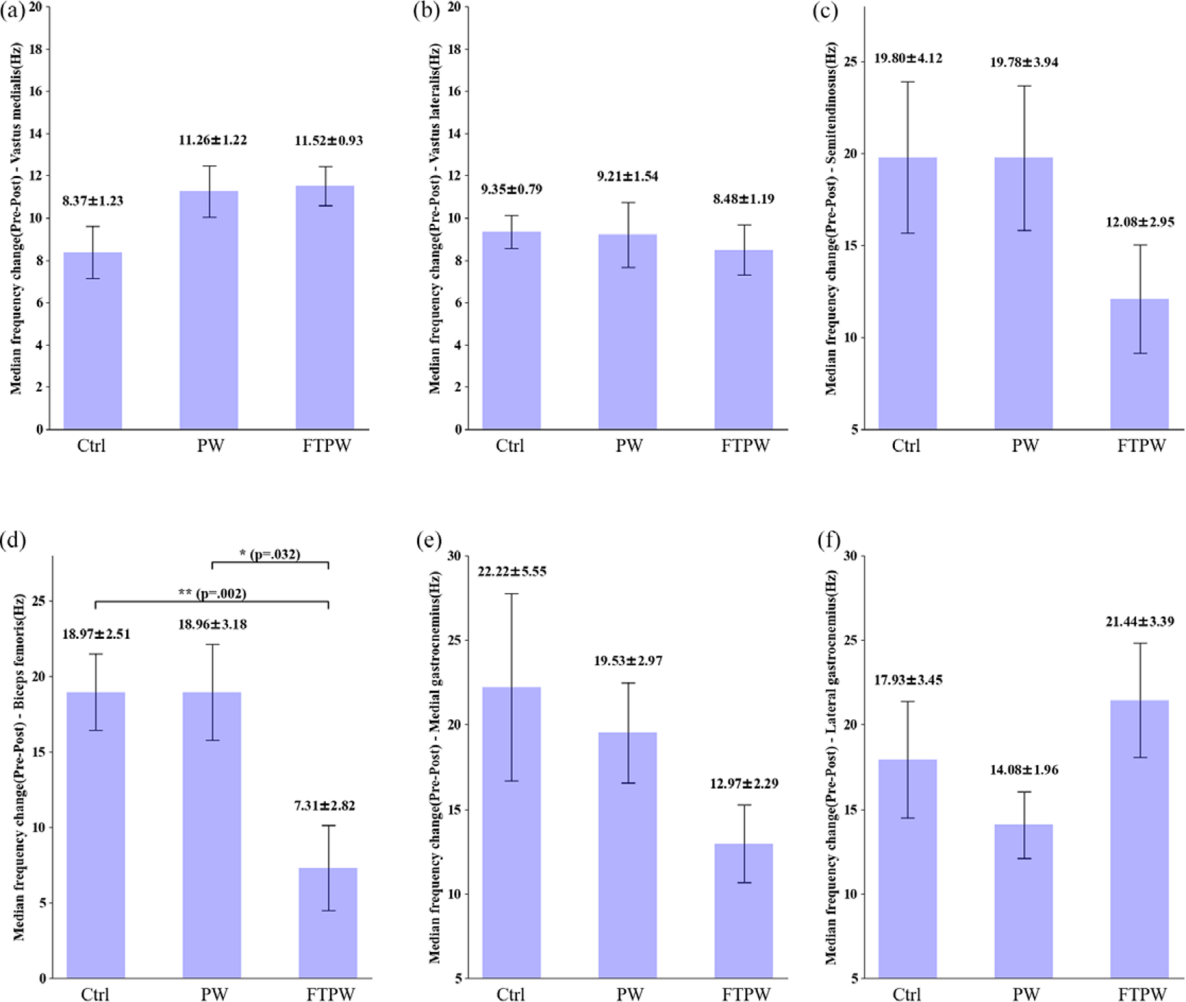
The median frequency difference of each muscle during the first and last 30 s of Climbmill exercise for the different conditions. Error bars mean standard error, and the value above the error bars is mean ± standard error. Asterisks (* and **) indicate statistically significant differences between clothing conditions (*p* < .05 and *p* < .01).

The results regarding changes in the median frequency of muscles are presented in (Figure 5). No significant differences between conditions were found in VM (*F*(1.62, 14.58) = 1.34, *p* = .29, *η*2 = .16, Figure 5a) and VL (*F* (2.00, 18.00) = .39, *p* = .68, *η*2 = .05, Figure 5b). Similarly, no significant differences between conditions were found in ST (*F* (1.19, 1.71) = .39, *p* = .59, *η*2 = .05, Figure 5c), MGT (*F* (2.00, 18.00) = .71, *p* = .51, *η*2 = .09, Figure 5e), and LGT (*F* (1.79, 16.11) = .25, *p* = .76, *η*2 = .03, Figure 5f). However, there was a significant difference between conditions in BF (*F* (1.56, 14.04) = 4.81, *p* = .04, *η*2 = .41, Figure 5d). Post-hoc analysis revealed that FTPW exhibited significantly less fatigue compared to PW (*p* = .032) and Ctrl (*p* = .002).

**Figure 6.**
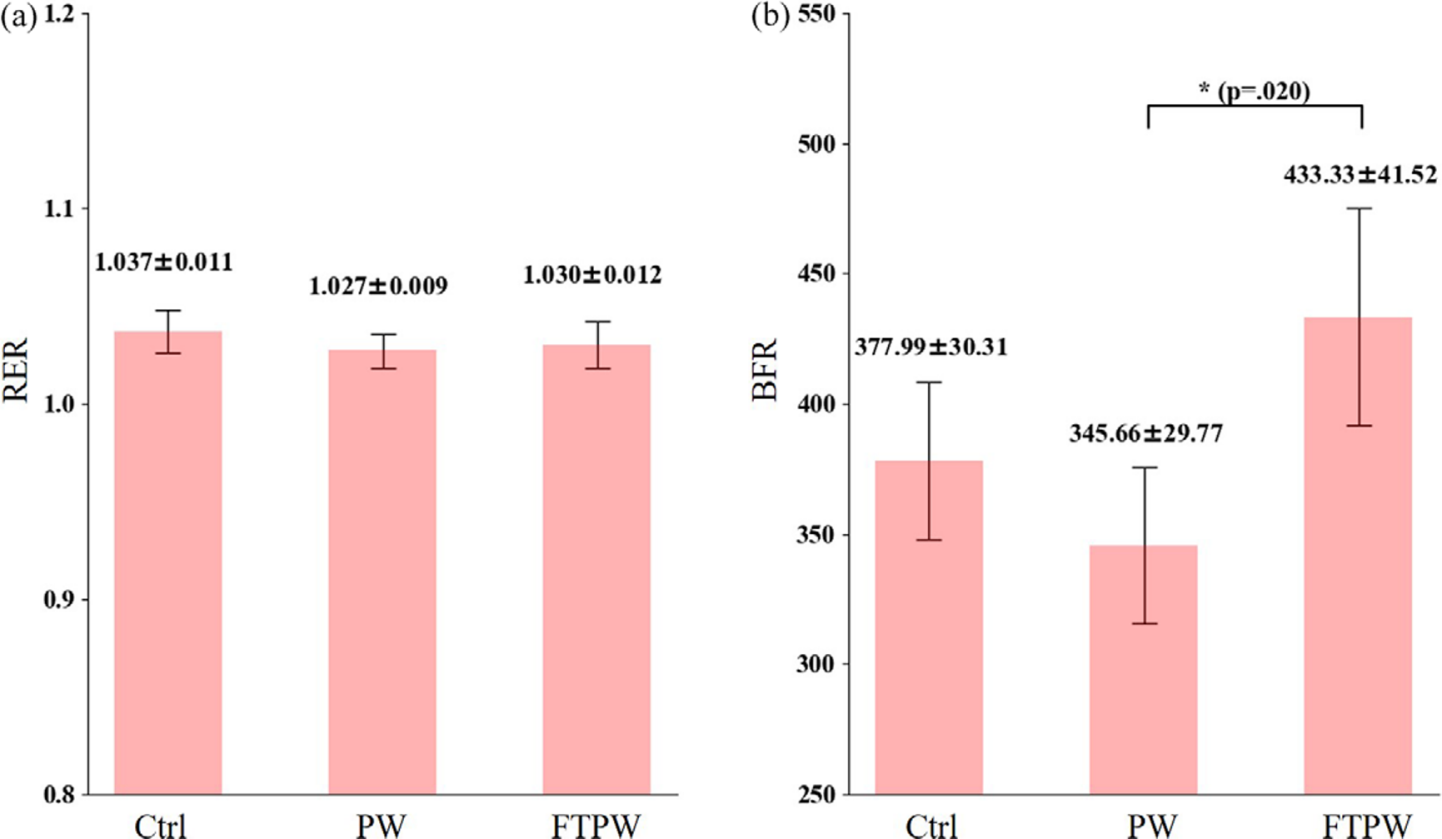
The RER (a) and BFR (b) for each condition. Error bars mean standard error, and the value above the error bars is mean ± standard error. Asterisk (*) indicates statistically significant differences between clothing conditions (*p* < .05).

Regarding metabolic cost analysis (Figure 6), no differences were found between conditions regarding the RER (*F* (1.463, 13.165) = 1.34, *p* = .290, *η*2 = .13, Figure 6a). The analysis of BFR showed statistically significant differences between conditions with Ctrl, PW, and FTPW (*F* (2.00, 12.00) = 6.54, *p* = .010, *η*2 = .52, Figure 6b). According to the results of the post-hoc analysis, there was a statistically significant increase in BFR in FTPW compared to PW (*p* = .020).

Regarding the subjective assessment (Table 1), paired *t*-tests revealed no significant differences between conditions for wearability (*p* = .538) or overall comfort (*p* = .608). Perceived compression, however, was significantly higher in the FTPW condition compared with PW (*p* = .022).

**Table 1.** Subjective assessment scores for the PW and FTPW conditions

	PW	FTPW	*p*-value
Wearability (0–20)	14.55 (4.28)	14.05 (3.09)	.538
Compression (0–25)	15.30 (4.17)	18.20 (3.25)	.022*
Comfort (0–25)	20.45 (4.15)	19.85 (2.87)	.608

*Note.* Values are presented as mean ± SD. Asterisks (*) denote statistically significant differences between clothing conditions (*p* < .05).

## 4. Discussion

The objective of this study was to investigate the knee injury prevention effects of combining passive wearable devices with knee flexion taping. The knee flexion angle at landing was significantly greater in the FTPW compared to both the PW and Ctrl in the post-fatigue SLD test. Importantly, increasing the knee flexion angle when landing is a known strategy to reduce injury risk (Leabeater et al., 2022). Accordingly, the FTPW’s ability to promote a soft landing can reduce the risk of a fatigued knee injury by distributing impact forces at the knee. Consistent with this kinematic difference, we observed lower sagittal-plane loading at the knee joint, together with a corresponding reduction in ground-reaction forces. This aligns with previous research showing that adequate knee flexion significantly reduces GRF and ACL loading during high-impact activities, thereby potentially mitigating injury risk (Decker and Ziegler, 2003; Kernozek et al., 2005; Kulas et al., 2008; Benjaminse et al., 2015; Beaulieu et al., 2023). This finding is particularly important because women typically exhibit reduced knee flexion and higher valgus angles upon landing, factors that contribute to their higher ACL injury rates (Beutler et al., 2009). By counteracting this tendency and enabling a deeper, soft landing, the FTPW may help lower the risk of knee injury in this population. Taken together, the FTPW’s enhancement of knee flexion and the reduction in joint loading demonstrated here in female participants directly address female-specific risk (McLean et al., 2005). The ability of FTPW to facilitate greater knee flexion while attenuating force and moment suggests its potential for injury prevention in sports and its applicability in rehabilitation, where controlled knee flexion is crucial for recovery.

In the frontal plane, the FTPW exhibited significantly greater knee varus than the PW and Ctrl during the SU test in both pre- and post-fatigue tests. Some studies have suggested that risk factors measured in the frontal plane, such as the angle and moment of knee joint valgus during landing, can lead to increased knee joint loading upon ground contact (Kar and Quesada, 2013; Chen et al., 2022). In addition, there is evidence that the risk of knee injury rises with a greater valgus angle (Shin et al., 2009), which increases lateral knee contact pressure (Levinger et al., 2013). Additionally, valgus loading and internal tibial rotation coupled with valgus motion were identified as contributing factors in the ACL injury mechanism (Nessler et al., 2017). By contrast, a moderate increase in varus motion can serve a protective role by distributing loads more medially and reducing excessive lateral compartment stresses, potentially mitigating ACL injury risk under certain conditions. This mechanism may be particularly relevant in activities such as the SU test, where frontal plane control is crucial for joint stability. Thus, the controlled increase in knee varus angle induced by the FTPW during the SU test may help stabilize the joint and protect it from injury, especially when muscles are fatigued and neuromuscular control is compromised.

However, it is noteworthy that excessive varus alignment of the knee can have negative implications for the prevention of osteoarthritis (OA). OA frequently manifests in the medial compartment of the knee, which experiences increased loading during knee varus (Knoop et al., 2012). Subjects exhibiting varus knee patterns should not undergo excessive correction but rather be maintained within a moderate range. The application of I-taping in the lateral direction proved effective in reducing excessive varus alignment in individuals with high knee varus patterns, and this effect can be further customized based on individual needs by adjusting the taping’s stiffness.

In addition to kinematic analysis, it was possible to analyze the effects on muscle activity, metabolic cost, and cardiovascular system caused by wearing FTPW. The change in median frequency of the BF was smaller when wearing the FTPW than when wearing other garments during the fatigue protocol. As fatigue increases, the median frequency decreases, and a small change in median frequency can be interpreted as a smaller decrease in fatigue (Ament et al., 1993). Regarding muscle activity, muscle fatigue is a concern, as it can impair the muscles’ capacity to generate strength (Lattier et al., 2004) and can lead to altered neuromuscular control (McLean et al., 2007). These factors can subsequently induce abnormal lower limb biomechanics, thereby increasing the risk of knee injury (Santamaria and Webster, 2010). In particular, the BF long head is heavily affected by fatigue, involving around 80% of all hamstring strain injuries (Bourne et al., 2016). Although the fatigue reduction was only significant for BF, other knee flexors – ST and MGT – also showed considerable reductions in fatigue when participants wore the FTPW. Therefore, the FTPW can help injury prevention of the lower extremities by reducing fatigue in the knee flexors.

We observed a notable increase in BFR when participants wore the FTPW. This elevation in BFR aligns with findings from a previous study, where increases when using passive wearable devices were consistently noted (Palya and Kiss, 2020). This phenomenon is indicative of a warming effect on the lower body (Leabeater et al., 2022). Furthermore, blood flow increases due to the compression of clothing, which reduces fatigue through oxygenation of muscles and removal of metabolites (Sperlich et al., 2013). These factors influence the effectiveness of passive wearable devices used in sports. It is expected that it will be effective when worn for a long time, as shown in previous studies (Lee et al., 2021).

As a result of metabolic cost analysis, there was no statistically significant difference between clothing conditions. The study implemented a fatigue protocol using the Climbmill, which necessitated the sustained maintenance of a significant knee flexion angle. Therefore, due to the reduction in fatigue among the knee flexors, such as BF, it is likely that this led to a subsequent decrease in metabolic cost. However, in line with the findings of a previous study (Lovell et al., 2011), the wearing of passive wearable devices led to an increase in the RER, implying a potential trade-off effect between reduced fatigue and the compression effect.

The subjective assessment indicated that wearability and overall comfort did not differ between devices, whereas the FTPW elicited a stronger sensation of compression, particularly around the thigh and shank regions (Supplementary Table S1). Although participants reported this heightened pressure, the accompanying differences in comfort and tactile perception were minimal. This implies that the biomechanical benefits demonstrated by the FTPW were not compensated by additional discomfort. In addition, some of the study participants with prior experience on conventional knee braces gave subjective comments that the FTPW provided similar landing stability while allowing greater freedom of movement or comfort.

The FTPW’s elastic, body-conforming design lets athletes wear it during training or daily routines without restricting natural movement. Like standard compression garments, it is lightweight and flexible, allowing it to be incorporated into uniforms without affecting performance. By increasing knee flexion at landing, the device promotes a soft-landing strategy that lowers impact forces and injury risk in jump-intensive sports. It is also reusable, easy to apply without professional help, and less likely to irritate the skin compared to kinesio tape. These advantages make the FTPW a practical support option that offers both protection and mobility for dynamic athletic activities. However, the FTPW’s support pattern is sewn into the fabric and cannot be reoriented to suit day-to-day needs. For example, if an athlete develops quadriceps soreness and needs taping that facilitates knee extension, the current design may not offer the desired assistance. Nevertheless, for most recreational users, it still provides meaningful knee stabilization during regular exercise.

There are a few limitations in this study. The FTPW is prototyped in the laboratory, and two sizes of FTPW were used for all subjects; therefore, the intended taping patterns might not have been perfectly aligned for some subjects. Moreover, our findings are based solely on the SU and SLD tasks. Additional assessments, such as running and jump landings under various load conditions, are needed to generalize the efficacy of the FTPW to broader athletic or daily-life scenarios. Despite these limitations, the present study underscores the promise of integrating targeted elastic taping into passive wearable devices to enhance knee flexion and minimize injury risks, particularly under fatigue-induced conditions.

## 5. Conclusion

This study showed that the FTPW was mainly effective in promoting safer knee joint kinematics in both the frontal plane and sagittal plane. Furthermore, the FTPW exhibited biological effects resulting from knee flexion assistance and compression. These results provide an encouraging outlook for the future development of passive wearable devices for injury prevention.

## Supporting information

Park et al. supplementary material Park et al. supplementary material

## Data Availability

Data can be made available to interested researchers upon request by email to the corresponding author.

## Supplementary material

The supplementary material for this article can be found at https://doi.org/10.1017/wtc.2025.10022.
