# The effect of body-conforming passive wearable device with knee flexion taping on dynamic knee stability – CORRIGENDUM

**DOI:** 10.1017/wtc.2025.10031

**Published:** 2025-09-18

**Authors:** Sung-Jin Park, Seongok Chae, Hyung-Soon Park

The authors regret that an incorrect reference was included in this published article. All references to “Sperlich et al. 2013” should instead have read “O’Riordan et al. 2023”. The full reference is provided below.
