# Supplementary material for: The effect of body-conforming passive wearable device with knee flexion taping on dynamic knee stability
Source: Wearable Technol. 2025 Aug 26;6:e43. doi: 10.1017/wtc.2025.10022 (PMC12441638; doi:10.1017/wtc.2025.10022)
Supplement: Park et al. supplementary material [file S2631717625100224sup001.docx]

**Supporting Information**

**List of Contents:**

**Supplementary Figure S1.** Knee joint rotation during the SLD/SU test before the fatigue protocol in low varus group.

**Supplementary Figure S2.** Knee joint rotation during the SLD/SU test before the fatigue protocol in high varus group.

**Supplementary Figure S3.** Knee joint rotation during the SLD/SU test after the fatigue protocol in low varus group.

**Supplementary Figure S4.** Knee joint rotation during the SLD/SU test after the fatigue protocol in high varus group.

**Supplementary Figure S5.** Knee joint moment/force during the SLD test after the fatigue protocol.

**Supplementary Figure S6.** Peak vGRF during SLD test

**Supplementary Table S1.** Subjective assessment detailed scores for the PW and FTPW conditions.


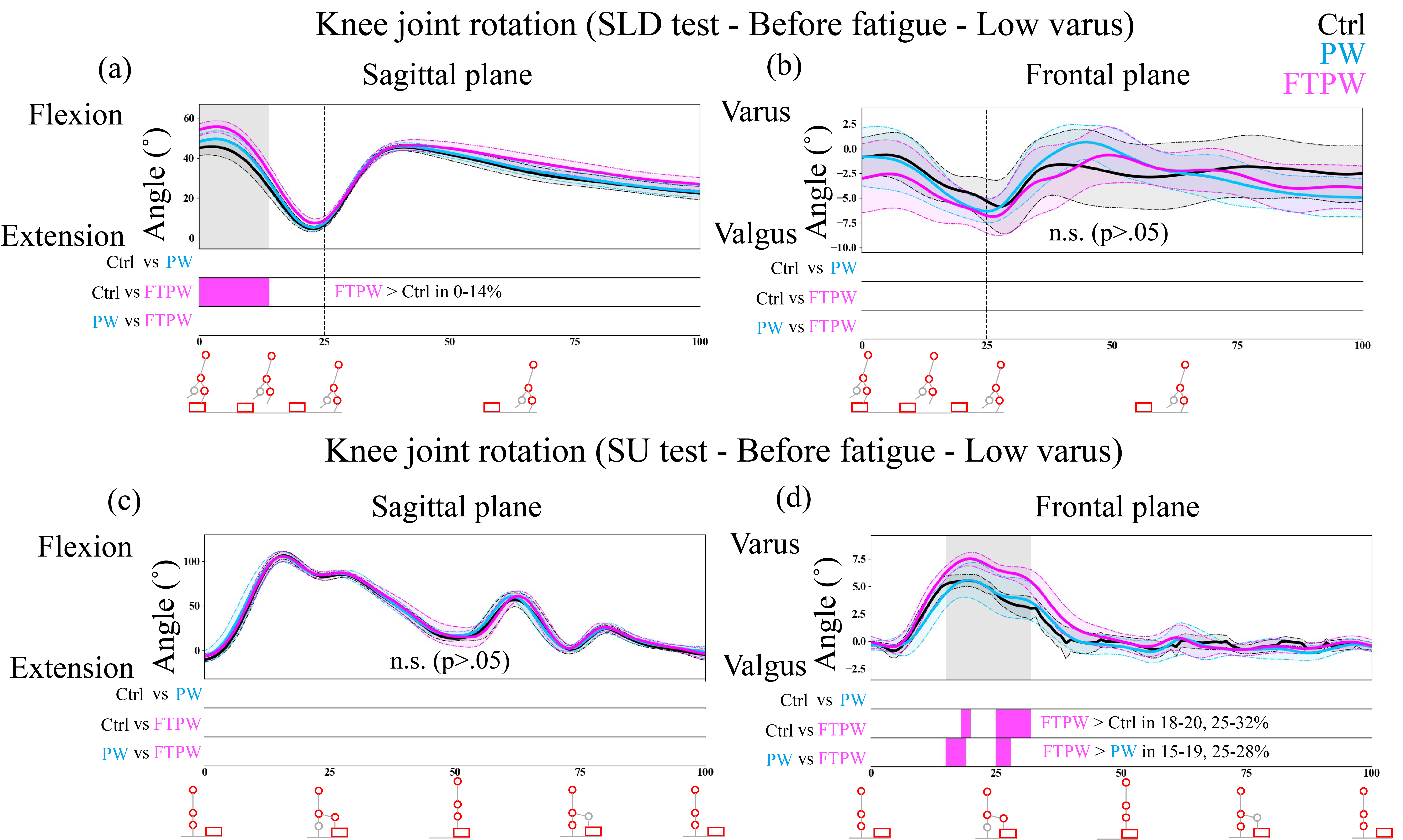


| **Figure S1.** **Knee joint rotation during the SLD/SU test (A, B/C, D) before the fatigue protocol in low varus group.** The black, blue and magenta line represent Ctrl, PW and FTPW, respectively. The gray-shaded area corresponds to sections where the repeated measures ANOVA results indicate a statistically significant difference (*p* < .05). Below each graph, bars compare group differences at each time point: Ctrl vs. PW, Ctrl vs. FTPW, and PW vs. FTPW. The color of bars corresponds to the group with larger values.  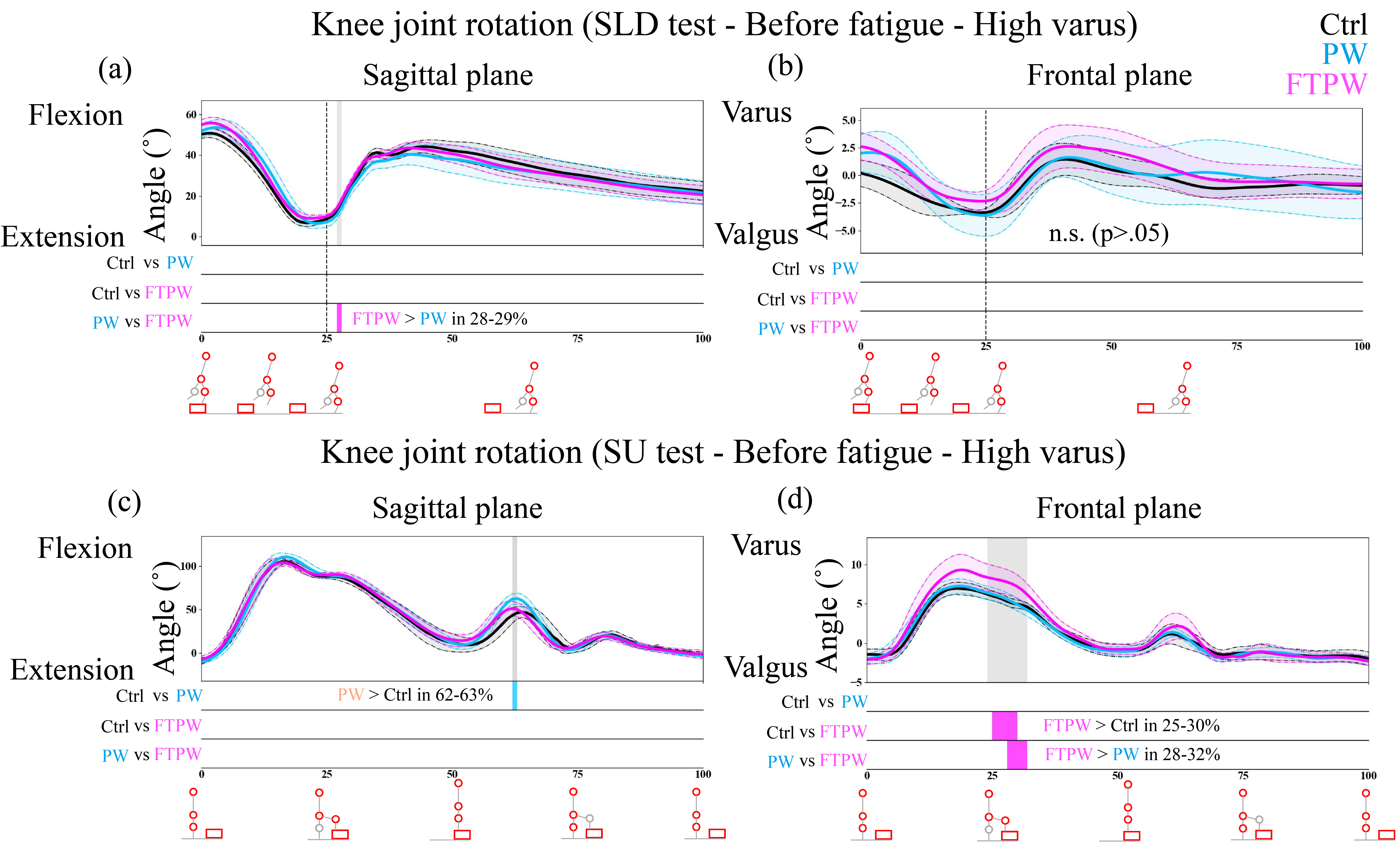  **Figure S2. Knee joint rotation during the SLD/SU test (A, B/C, D) before the fatigue protocol in high varus group.** The black, blue and magenta line represent Ctrl, PW and FTPW, respectively. The gray-shaded area corresponds to sections where the repeated measures ANOVA results indicate a statistically significant difference (*p* < .05). Below each graph, bars compare group differences at each time point: Ctrl vs. PW, Ctrl vs. FTPW, and PW vs. FTPW. The color of bars corresponds to the group with larger values.  **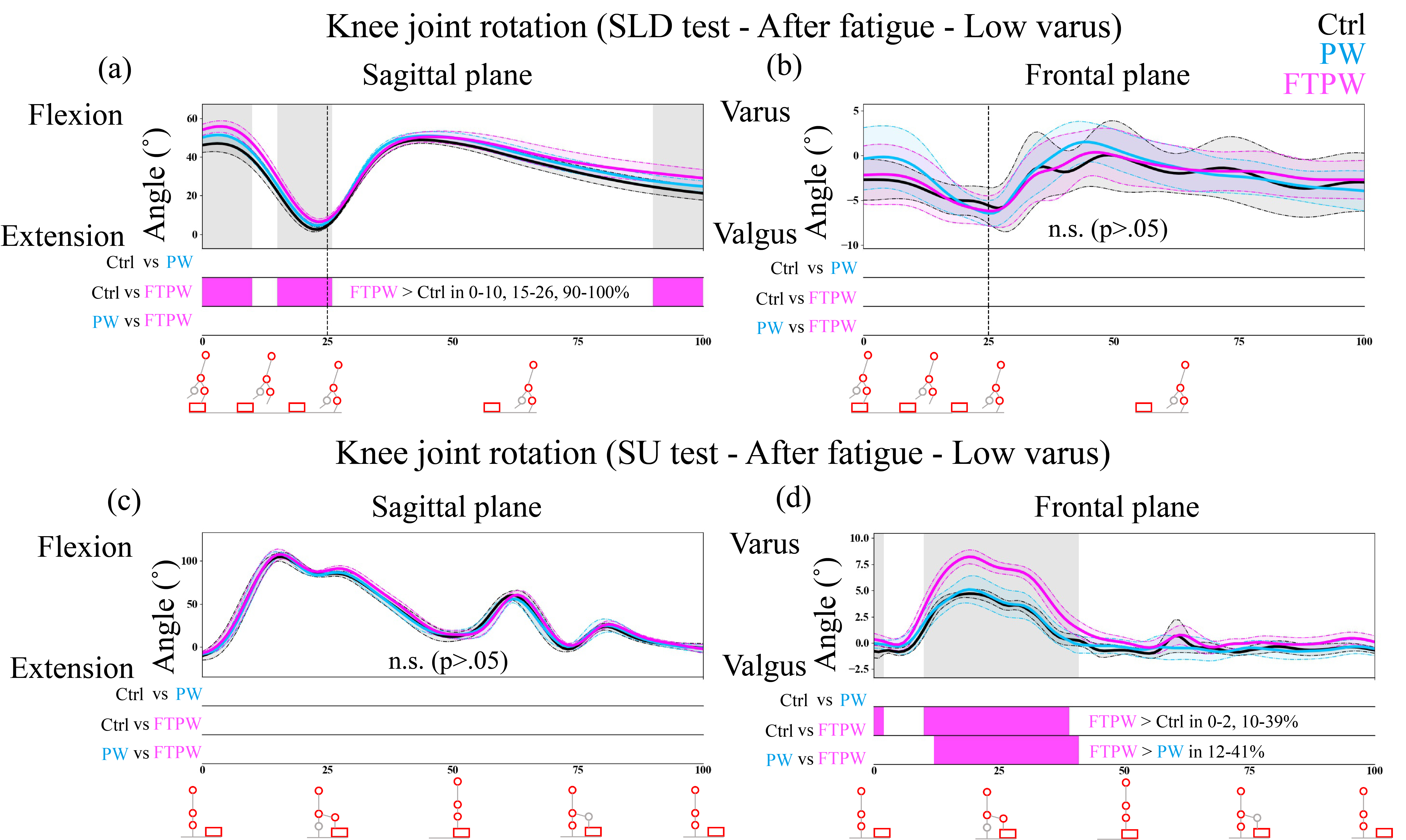Figure S3. Knee joint rotation during the SLD/SU test (A, B/C, D) after the fatigue protocol in low varus group.** The black, blue and magenta line represent Ctrl, PW and FTPW, respectively. The gray-shaded area corresponds to sections where the repeated measures ANOVA results indicate a statistically significant difference (p < .05). Below each graph, bars compare group differences at each time point: Ctrl vs. PW, Ctrl vs. FTPW, and PW vs. FTPW. The color of bars corresponds to the group with larger values.  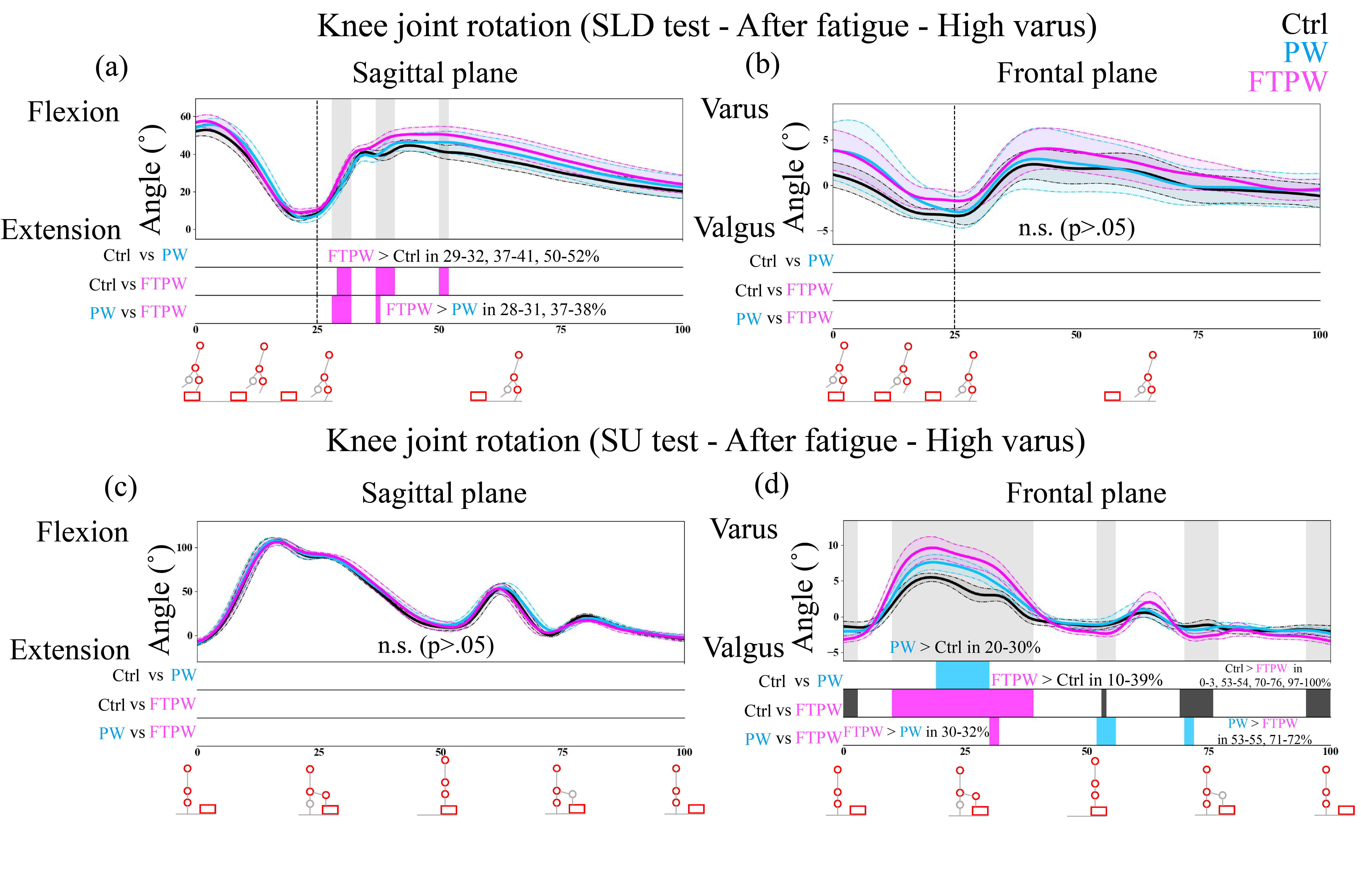  **Figure S4. Knee joint rotation during the SLD/SU test (A, B/C, D) after the fatigue protocol in high varus group.** The black, blue and magenta line represent Ctrl, PW and FTPW, respectively. The gray-shaded area corresponds to sections where the repeated measures ANOVA results indicate a statistically significant difference (p < .05). Below each graph, bars compare group differences at each time point: Ctrl vs. PW, Ctrl vs. FTPW, and PW vs. FTPW. The color of bars corresponds to the group with larger values. |
| --- |


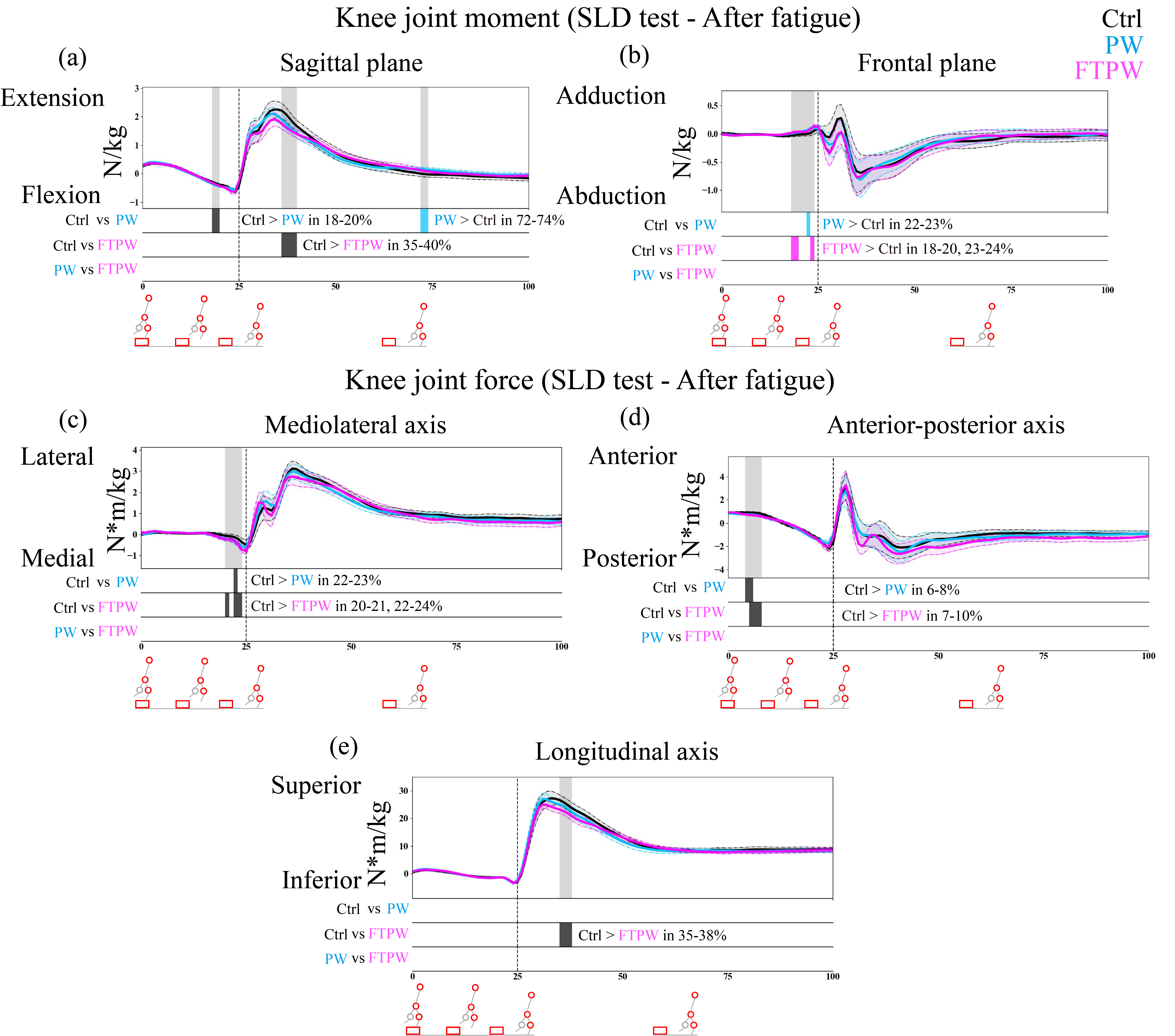


**Figure S5.** **Knee joint moment/force during the SLD test (A, B/C, D, E) after the fatigue protocol.** The black, blue and magenta lines represent Ctrl, PW, and FTPW, respectively. Error bars mean standard error. The gray-shaded area corresponds to sections where the repeated measures ANOVA results indicate a statistically significant difference (p < .05). Below each graph, bars compare group differences at each time point: Ctrl vs. PW, Ctrl vs. FTPW, and PW vs. FTPW. The color of bars corresponds to the group with larger values.


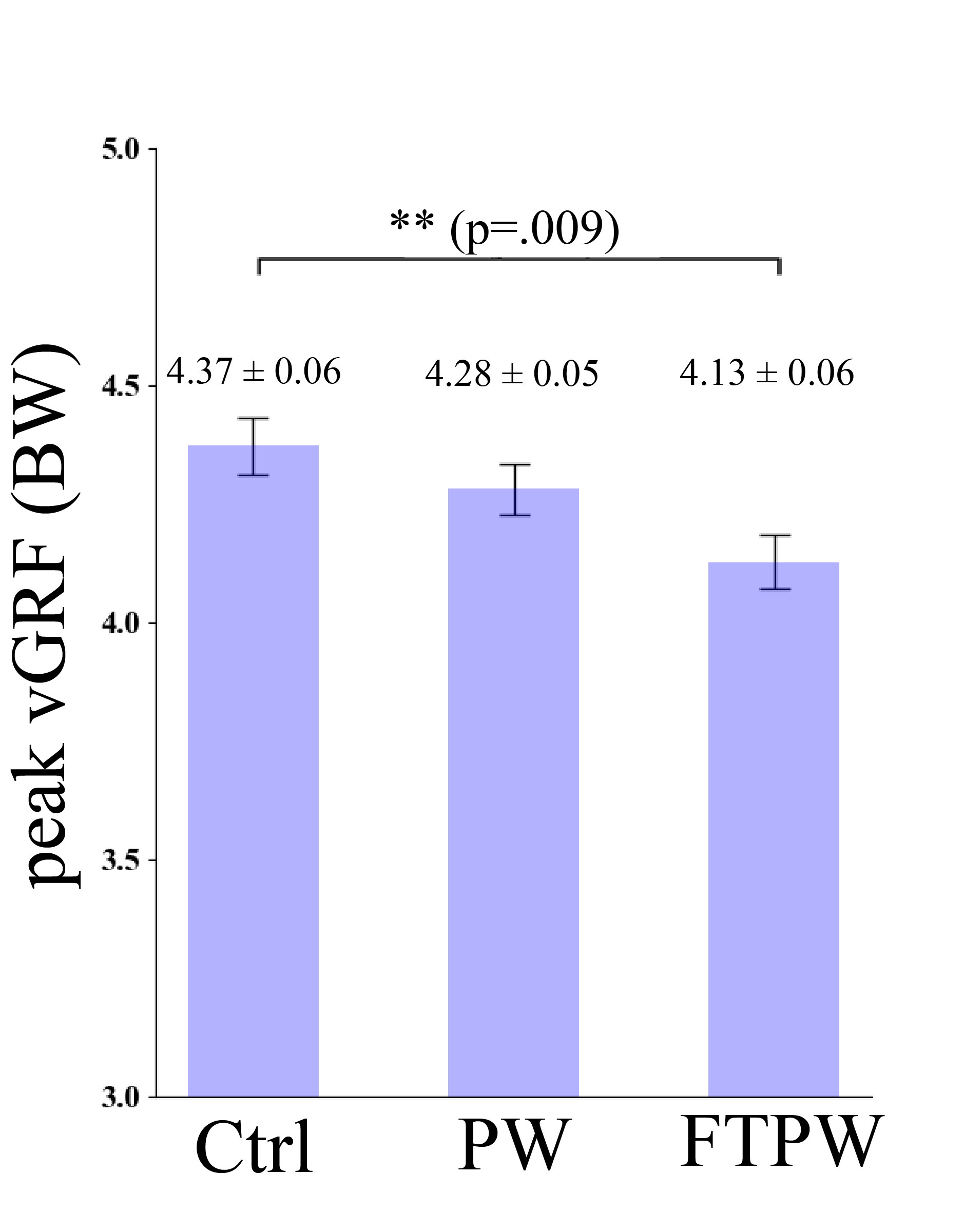


**Figure S6**. **Peak vGRF during SLD test for each condition. Error bars mean standard error, and the value above the error bars is mean ± standard error.** Asterisk (**) indicates statistically significant differences between clothing conditions (p < .05). there was a significant difference between conditions in peak vGRF (F (0.64, 3.76) = 4.85, p = .01). Post-hoc analysis revealed that FTPW exhibited significantly less peak vGRF to Ctrl (p = .0089).

|  | | PW | FTPW | P-value |
| --- | --- | --- | --- | --- |
| Wearability | Length | 3.55 (1.10) | 3.60 (0.99) | .853 |
|  | Thigh | 3.75 (1.12) | 3.85 (0.99) | .705 |
|  | Shank | 3.65 (1.09) | 3.00 (1.07) | .015* |
|  | Waist | 3.60 (1.14) | 3.60 (0.99) | 1.000 |
| Comfort | Breathing | 4.20 (0.77) | 4.15 (0.59) | .825 |
|  | Resting | 4.05 (0.89) | 3.95 (0.76) | .715 |
|  | Squating | 3.95 (0.89) | 3.75 (0.72) | .463 |
|  | Sitting | 4.15 (0.99) | 3.80 (0.83) | .232 |
|  | Walking | 4.10 (1.02) | 4.20 (0.77) | .693 |
| Compression | Overall | 3.45 (1.15) | 4.05 (0.94) | .103 |
|  | Abdomen | 3.00 (1.26) | 2.85 (1.04) | .720 |
|  | Buttocks | 3.15 (1.23) | 3.25 (0.78) | .776 |
|  | Thigh | 2.75 (1.02) | 3.90 (0.91) | <.001*** |
|  | Shank | 2.95 (1.05) | 4.15 (0.81) | <.001*** |

**Table S1. Subjective detailed assessment scores for the PW and FTPW conditions.** Values are presented as mean ± SD. Asterisks (*,***) denote statistically significant differences between clothing conditions (p < .05, p<.001).
